# Urgent and emergent radiotherapy for hematologic malignancies of the central nervous system: a review of the literature and practical approach

**DOI:** 10.3389/fonc.2025.1511261

**Published:** 2025-03-31

**Authors:** Kathryn R. Tringale, Brandon S. Imber, Gustav Y. Cederquist, Joachim Yahalom, Zachary R. Moore, Richard T. Hoppe, Michael S. Binkley, Jason B. Ross, N. Ari Wijetunga, Parag Sanghvi, Dana L. Casey, Susan M. Hiniker

**Affiliations:** ^1^ Department of Radiation Medicine and Applied Sciences, University of California San Diego, La Jolla, CA, United States; ^2^ Department of Radiation Oncology, Memorial Sloan Kettering Cancer Center, New York, NY, United States; ^3^ Department of Radiation Oncology, Stanford University, Palo Alto, CA, United States; ^4^ Department of Radiation Oncology, University of North Carolina, Chapel Hill, NC, United States

**Keywords:** central nervous system, lymphoma, leukemia, hematologic malignancies, radiotherapy, emergency, urgency

## Abstract

**Introduction:**

Hematologic malignancies, including leukemias, lymphomas, and myeloma, can involve the central nervous system (CNS) at the time of diagnosis or later in relapse. CNS involvement can lead to acute neurologic symptoms or signs that need prompt evaluation and treatment. Radiotherapy (RT) can lead to quick disease response, but how it can best be incorporated early into multi-modality treatment in the urgent clinical setting is often unclear.

**Methods:**

Here, we outline a practical approach to planning and incorporating urgent RT in patients with hematologic malignancies involving the CNS. We provide a review of the literature to inform RT indications, timing, dosing, and treatment volumes by histology and clinical scenario. We also highlight evolving controversies in this field and growing indications for RT in conjunction with novel therapeutics.

**Results:**

RT is often the quickest-acting, most reliable tool to salvage cranial neuropathies or neurologic deficits and should be considered early. If systemic or intrathecal therapy are expected to achieve swift response as upfront treatment, simulation should still be planned in the event that response is delayed and RT is needed. RT in combination with certain systemic or intrathecal therapies can lead to unacceptable neurotoxicity; therefore, early multidisciplinary discussion to appropriately sequence therapies is critical. Thorough work-up with systemic imaging, complete neuroaxis MRI, ophthalmologic exam, and cerebrospinal fluid sampling can dictate target volumes from focal RT to comprehensive craniospinal irradiation (CSI). Dosing can range from as low as 4 Gray (Gy) for indolent disease to 36-50 Gy for more aggressive or refractory disease. Often, mid-treatment re-planning can be considered to address swift volume reduction to improve the therapeutic window. RT plays a promising role for bridging symptomatic patients to novel therapeutics (e.g., chimeric antigen receptor T-cell therapy), but optimal dosing and treatment volumes are evolving topics that require further prospective evaluation.

**Conclusions:**

RT is a powerful tool for achieving rapid responses in hematologic malignancies and therefore should be considered early in urgent neurologic settings. Thorough workup and discussions with the multi-disciplinary team are critical to best incorporate RT in the context of other CNS-penetrating therapies. Further work is warranted on defining RT target volumes in the context of novel therapeutics.

## Introduction

Patients with hematologic malignancies may develop neurologic signs or symptoms that require urgent evaluation for radiotherapy (RT) to preserve and restore neurologic function. It is key to evaluate the patient within the entire clinical context, including histology, neurologic status, goals of treatment, plans for current or future systemic therapy, and expected likelihood and timeframe of response to other potential therapies. These factors may vary widely depending on the patient’s clinical situation and treatment history. As the life expectancy of patients with hematologic malignancies increases with improvement in systemic therapies, we have learned more about patterns of disease failure involving the central nervous system (CNS) as a sanctuary site. CNS involvement presents a clinical challenge as various compartments of the neuraxis (e.g., parenchyma, leptomeninges, eyes) can be involved and treatments must penetrate the blood brain barrier (1). Here, we review the limited existing literature on this evolving topic and provide a practical approach to urgent RT based on expert opinion in hematologic malignances involving the CNS.

## Clinical considerations for CNS-directed radiation therapy

### Clinical work-up and data collection

First, a histologic diagnosis should be established through tissue sampling. In patients who had prior systemic involvement of disease and now present with secondary CNS lymphoma, it is important to biopsy the CNS-involved site to rule out alternate diagnoses or to identify transformation of a prior indolent lymphoma. In general, indolent hematologic malignancies may only require very low dose RT with substantial clinical benefit and minimal toxicity, while aggressive histologies may require higher doses.

Second, the anatomic distribution of disease should be established through a combination of physical exam, imaging, and tissue sampling. This evaluation includes neurologic and ophthalmologic exam with slit lamp, extracranial imaging and comprehensive MRI of the entire neuraxis, and finally consideration of vitreoretinal and/or cerebrospinal fluid (CSF) sampling for both cytology and flow cytometry. Extent of neuraxis involvement can dictate RT target volumes as well as inform the choice of systemic or intrathecal (IT) therapies (2).

Third, the status of the disease should be established (i.e. primary versus relapsed or refractory). Disease status can impact the radiation dose and treatment volume. For instance, in the setting of first-line therapy for primary CNS lymphoma, RT now typically plays a consolidative role; however, it can be considered as monotherapy in patients ineligible for systemic therapy. Yet for patients with refractory primary CNS lymphoma, the role of RT is broadened to palliative, bridging, or salvage therapy, often necessitating a higher dose to overcome refractoriness. Therefore, multidisciplinary discussion with involved team members (e.g., Hematology/Oncology, Neurology, and Cellular Therapy) is imperative. These discussions can also provide an estimate of likely time-to-response of systemic therapies, whether future therapies are available, or if RT is the only viable option. All of these factors can influence RT timing, volumes, and dosing.

Lastly, consideration of prior RT courses, both CNS- and systemically-directed treatments, can provide valuable information. Of course, consideration of volume overlap with and time interval from prior treatment to plan safe delivery of RT to organs-at-risk (OARs) should always be considered, but specifically understanding an individual patient’s disease response to prior RT can inform the prescription and anticipated outcome. For instance, in patients who had prior excellent response to RT to non-CNS-targeted lesions, it is reasonable to consider a similar dose for CNS disease. **Table 1** summarizes the key features to consider when planning RT within the context of multimodality treatments for these complex patients.

**Table 1:** Clinical considerations for CNS-directed radiation therapy.

Clinical considerations	
Histology	Primary CNS Lymphoma (Aggressive vs. Indolent) Secondary CNS Lymphoma (Aggressive vs. Indolent) Multiple Myeloma Leukemia
Anatomic Distribution	Vitreoretinal Parenchymal Leptomeningeal (Radiographic vs. Cytologic) Osseous (Spine vs. Calvarium/Base of Skull)
CNS Disease Status	Newly Diagnosed Relapsed/Refractory
Extracranial Disease Status	Controlled Uncontrolled
Intent of Treatment and Future Plans for Systemic or Intrathecal Therapy	Bridging (Temporal vs. Cytoreductive) Salvage Palliation
Prior Radiation Therapy	Dose Volume Overlap/Proximity Organ-At-Risk Tolerance Time Interval

### Rationale for consideration of radiation therapy in the urgent or emergent setting

Patients with cranial nerve abnormalities and/or other neurologic impairments are best treated with upfront RT, often in coordination with multi-modality systemic or IT therapies. For instance, in CNS leukemia, RT can lead to resolution or improvement of symptoms in ~70% patients, stability in ~15%, and progression of symptoms in ~15% (3). RT is a highly active CNS agent, rendering it often the quickest and most reliable way to salvage cranial and spinal nerves. One study of CNS-directed RT as a bridge to chimeric antigen receptor (CAR) T-cell therapy in CNS lymphoma showed a 74% mean reduction in tumor size from baseline at a median of 12 days from RT completion (4). RT should be considered as soon as symptomatic nerve involvement is suspected, given that the chance of nerve recovery declines with time from presentation.

### Care of the pediatric patient

First, it is important to acknowledge that pediatric patients have highly variable RT-associated toxicity risks depending on age (5). For instance, RT should be avoided if possible in the youngest patients (particularly for those under age 3), and instead a non-RT-containing approach of treating CNS disease should be considered based on multi-disciplinary discussion. Second, in addition to age, radiation dose and volume are independent predictors of neurocognitive decline, with a 5% risk of subsequent intelligence quotient (IQ) <85 if 50% of the brain receives 22.2 Gy or 100% of the brain receives 18.1 Gy (6). Third, use of chemotherapy in children, particularly methotrexate, can compound the effect on neurotoxicity, with the risk of IQ <85 similar to a uniform brain dose of approximately 6 Gy. While caution and awareness of toxicity is paramount in children, we must remember that many of these young patients with leukemia are highly curable, with 5-year relative survival rates for childhood leukemia of 86% (7). Therefore, we should only de-escalate therapy to reduce potential toxicity if we are certain that disease control will not be compromised.

### Logistical coordination with a multi-disciplinary team

#### Corticosteroid administration

Corticosteroids can rapidly improve neurologic symptoms and reduce the risk of complications. Radiographic reduction in mass size can occur in ~40% of patients with primary CNS lymphoma treated with corticosteroids (8); however, this finding is nonspecific for CNSL diagnosis and can actually delay or prevent the diagnosis in approximately 50% of patients if delivered prior to tissue sampling (9). Therefore, we recommend multi-disciplinary discussion regarding plans for biopsy prior to steroid administration.

#### Simulation scheduling

If a patient is treatment-naïve and there is felt to be a high chance of systemic therapy successfully (and swiftly) reversing neurologic deficits, the patient should be followed closely with an RT simulation ideally reserved to occur 48 hours after initiation of chemotherapy. This setup will allow the patient to start RT urgently if adequate clinical response to systemic therapy has not been achieved.

#### Overlap versus interdigitating with systemic or intrathecal therapy

If necessary, IT therapy can be considered in select cases 2-3 times per week along with focal RT; however, there is some risk with concurrent delivery and others prefer to avoid concurrent IT treatment with cranial RT entirely. Data in this scenario are limited, and therefore concurrent use is often avoided given concerns published in smaller series and extrapolated from solid malignances (10). Reported toxicities of concurrent administration of IT chemotherapy and involved-field RT include acute meningitis, chronic‐delayed encephalopathy, radiculitis, myelosuppression, and mucositis, with grade 3-4 adverse effects reported on the range of 15-55% (11–14). Notably, one prospective study of 59 patients showed two treatment-related deaths from encephalopathy and meningitis (13). When interdigitating treatments, the prolonged half-lives of these drugs should be considered: up to 24 hours for IT cytarabine and biphasically up to 4.5 and 14 hours for IT methotrexate (but up to 44 hours in patients with renal impairment) (15). Notably, both drugs can have further increased CSF levels when given with systemic administration concurrently. In general, high-dose CNS-penetrant intravenous (IV) systemic therapy (e.g., methotrexate, cytarabine) should be avoided for two weeks before or after cranial RT, although this interval depends on the clinical situation and extent of RT field (3, 16). For instance, a smaller interval of 48-72 hours can be considered if urgent RT is needed, especially with focal spine RT as opposed to cranial targets (16). Regardless of decision regarding systemic or IT therapy, RT should take precedence for treatment of acute neurologic symptoms.

#### Radiation planning

Many hematologic malignancies are radiosensitive and therefore prompt and significant target volume change can occur even after several fractions. Therefore, physicians should pay close attention to on-beam imaging (with consideration for daily cone-beam CT for bulky base of skull or spine tumors) to assess whether a re-plan may be indicated. A re-plan should be strongly considered in the setting of radiosensitive disease adjacent to sensitive OARs. If feasible to be prepared promptly, intensity-modulated radiation therapy (IMRT), proton therapy, or other conformal techniques are often preferred for bone marrow and normal tissue sparing, which can also help limit OAR dosing for marrow preservation and future courses of RT often needed in hematologic malignancies. When targeting the eyes, coverage of the at-risk sites (e.g., posterior and/or anterior chamber) should be the focus and take precedence over sparing the lenses to avoid future cataracts. If there is particular concern regarding lens dose, the patient can be asked to maintain a fixed gaze at time of simulation and treatment so that daily setup is consistent to allow for more certainty of dose delivery to the lens. Regardless, patients should be counseled for risk of cataracts.

Some patients with symptomatic hydrocephalus or increased intracranial pressure may require ventriculoperitoneal shunt or large volume lumbar puncture. A repeat MRI of the brain should be considered after these interventions and prior to RT planning given possible changes in anatomy (especially if a focal RT field is planned). The radiation oncologist and RT planning team should also be aware of the material used for any implanted devices; however, these devices are often plastic and dosimetry does not need to be altered. Although radiation can interfere with re-epithelialization and wound healing after surgical procedures, delaying RT to allow for optimal wound healing may not be feasible or advisable in these urgent clinical scenarios. Instead, attention should be paid to patients with surgical incisions that lie within the RT field so that skin dose can be kept as low as reasonably achievable. At the time of simulation, we recommend wiring the site of surgical incision so that it can be used as an avoidance structure when planning beam entry angles and to minimize dose with scalp-sparing techniques (i.e., volumetric modulated arc therapy [VMAT]) (17).

## Histology-specific practical approach to radiation treatment planning in the urgent setting

A summary of consensus treatment recommendations for RT dose and target volume depending on histology and clinical status as described in the text below is summarized in **Table 2**.

**Table 2:** RT target approach by anatomic compartment involvement and histology.

Anatomic compartment	Histology	Disease status	Target	Dose
Focal parenchymal mass	PCNSL Aggressive SCNSL Indolent SCNSL	Refractory, not bridging Higher KPS Poor KPS Refractory, bridging intent Extracranial controlled Extracranial uncontrolled Extracranial controlled Extracranial uncontrolled	WBRT [boost] WBRT Focal WBRT [boost] Focal vs. WBRT Focal vs. WBRT Focal vs. WBRT	23.4Gy-36Gy/13-18fx [36-45Gy/20-25fx] 30Gy/10fx 30-33Gy/10-11fx 23.4Gy/13fx [36Gy/20fx] 30Gy/10fx [36Gy/20fx] 4Gy/2fx, 24Gy/12fx 4Gy/2fx, 24Gy/12fx
Dural-based disease	Marginal zone		Focal	4-24Gy/2-12fx
Cranial nerve deficit	Multiple myeloma (base of skull or calvarium)		Focal	24Gy/12fx 20Gy/5fx 30Gy/10fx (8Gy/1fx, least preferred)
Vitreoretinal	PVRL SCNSL		Bilateral globes and optic tracts Bilateral globes and optic tracts	30.6-36Gy/17-20fx 24Gy-36Gy/12-18fx
Spinal cord or cauda equina compression	Multiple myeloma Solitary plasmacytoma		Focal (full vertebral level involved) Focal (full vertebral level involved)	24Gy-30Gy/12-15fx 30Gy/10fx (4-8Gy/1fx, 20Gy/5fx both less preferred) 36-50Gy/18-25fx
Symptomatic leptomeningeal disease	PCNSL SCNSL Multiple myeloma		IFRT vs. CSI IFRT vs. CSI IFRT vs. CSI	30Gy/10fx 20-24Gy/10-12fx, 30Gy/10fx 20-24Gy/10-12fx, 30Gy/10fx
Leptomeningeal disease prior to hematopoietic cell transplantation^a^	Leukemia CNS cleared or CNS 1 T-cell ALL CNS persistent or radiographic LMD or cranial neuropathies Skeletally mature Skeletally immature		CSI vs. CSI [WBRT]^b^ CSI^c^ CSI [WBRT]^d^	18Gy/9fx vs.12Gy/6fx [18Gy/9fx] 18-24Gy/9-12fx 18Gy/9fx [24Gy/12fx]

Cumulative sequential boost shown in brackets.

PCNSL, primary CNS lymphoma; SCNSL, secondary CNS lymphoma; PVRL, primary vitreoretinal lymphoma; WBRT, whole-brain radiotherapy; IFRT, involved field radiotherapy; CSI, craniospinal irradiation; fx, fraction; LMD, leptomeningeal disease; TBI, total body irradiation.

a Dosing shown in this section is cumulative dose to the neuraxis, including TBI typically of 12 Gy in 6 fractions.

b A cumulative dose of 18 Gy WBRT is typically delivered (i.e., 6 Gy WBRT boost accounting for TBI dose typically of 12 Gy).

c For skeletally mature patients with radiographic leptomeningeal disease or cranial neuropathies at diagnosis or relapse, our general practice is to add 6-12 Gy CSI in addition to TBI (for a cumulative of 18-24 Gy to the craniospinal axis).

d For skeletally immature patients, the brain is typically boosted to an additional 12 Gy (24 Gy total), while the spine receives a 6 Gy boost (18 Gy total).

### Primary CNS lymphoma

Primary CNS lymphoma (PCNSL) is a rare CNS malignancy with systemic staging (i.e., PET/CT) negative for extracranial involvement. Historically, PCNSL was treated with RT alone, but nearly all patients experienced in- and out-of-field progression even when using doses as high as 60 Gray (Gy) (18). Consolidative RT after methotrexate-based chemotherapy can be curative, but to date, two randomized trials (IELSG32 and PRECIS) have shown cognitive decline with whole-brain doses of 36-40 Gy compared to autologous hematopoietic cell transplant (AHCT) (19, 20). Therefore, AHCT is now preferred for consolidation over standard-dose WBRT (i.e., >24 Gy) for fit patients who are eligible, although the higher treatment-related mortality must be noted. There is retrospective evidence that lower doses of consolidative radiotherapy (23.4 Gy in 13 fractions) after induction chemotherapy for complete responders may be sufficient and safe; however, the trend in management currently still favors AHCT and these approaches have never been prospectively compared. (21) Consolidative reduced-dose WBRT (23.4 Gy in 13 fractions) followed by cytarabine is a reasonable alternative if the patient is not a candidate for AHCT (21, 22). Non-myeloablative chemotherapy (e.g., cytarabine) can also be considered for consolidation, although preliminary data suggest possible reduced progression-free survival compared to reduced-dose WBRT (22).

**Figure 1:**
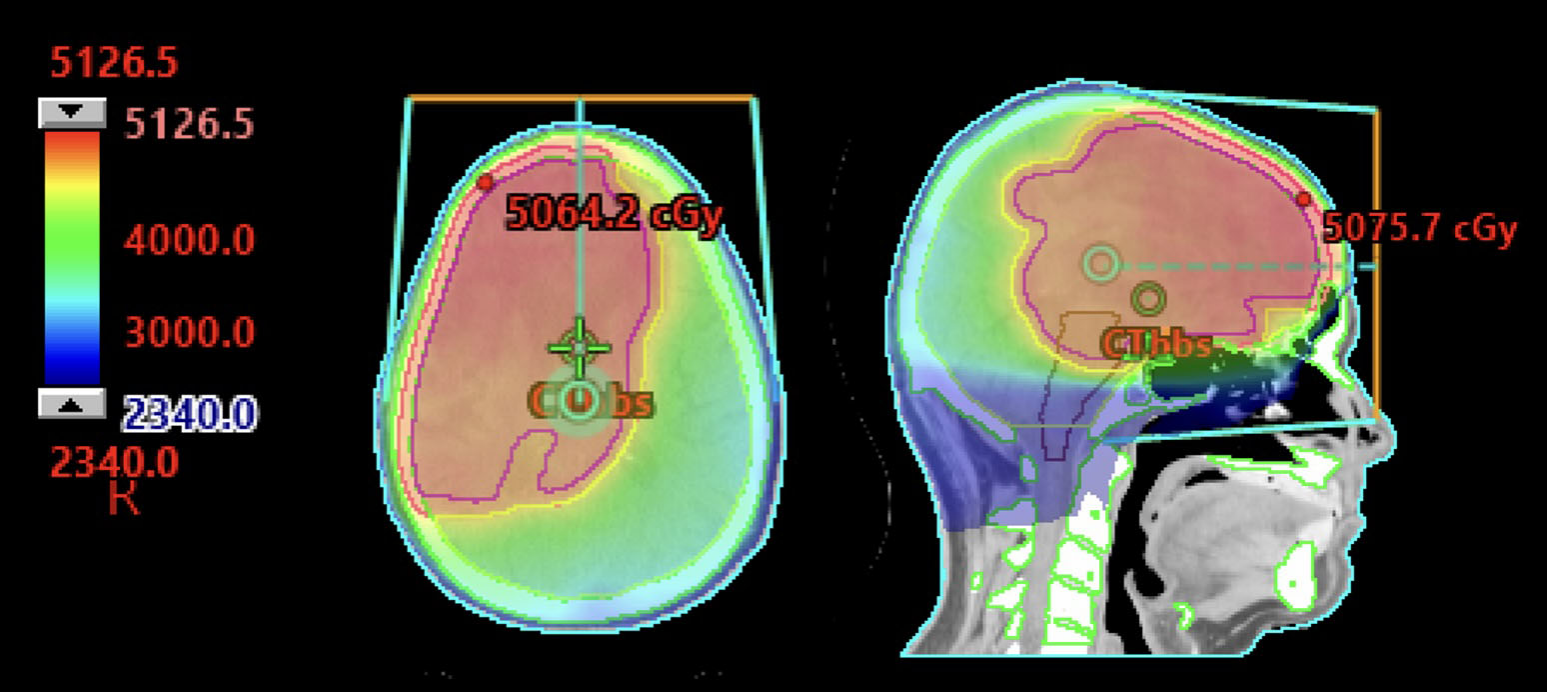
63-year-old immunocompetent man with primary CNS lymphoma treated per CALGB 50202 protocol with methotrexate, temozolomide, and rituximab with partial response followed by autologous hematopoietic cell transplant. He had an early recurrence 4 months later with negative lumbar puncture and PET/CT. He was treated with 23.4 Gy in 13 fractions to the whole brain with a sequential boost to a total of 45 Gy in 25 fractions.

**Figure 2:**
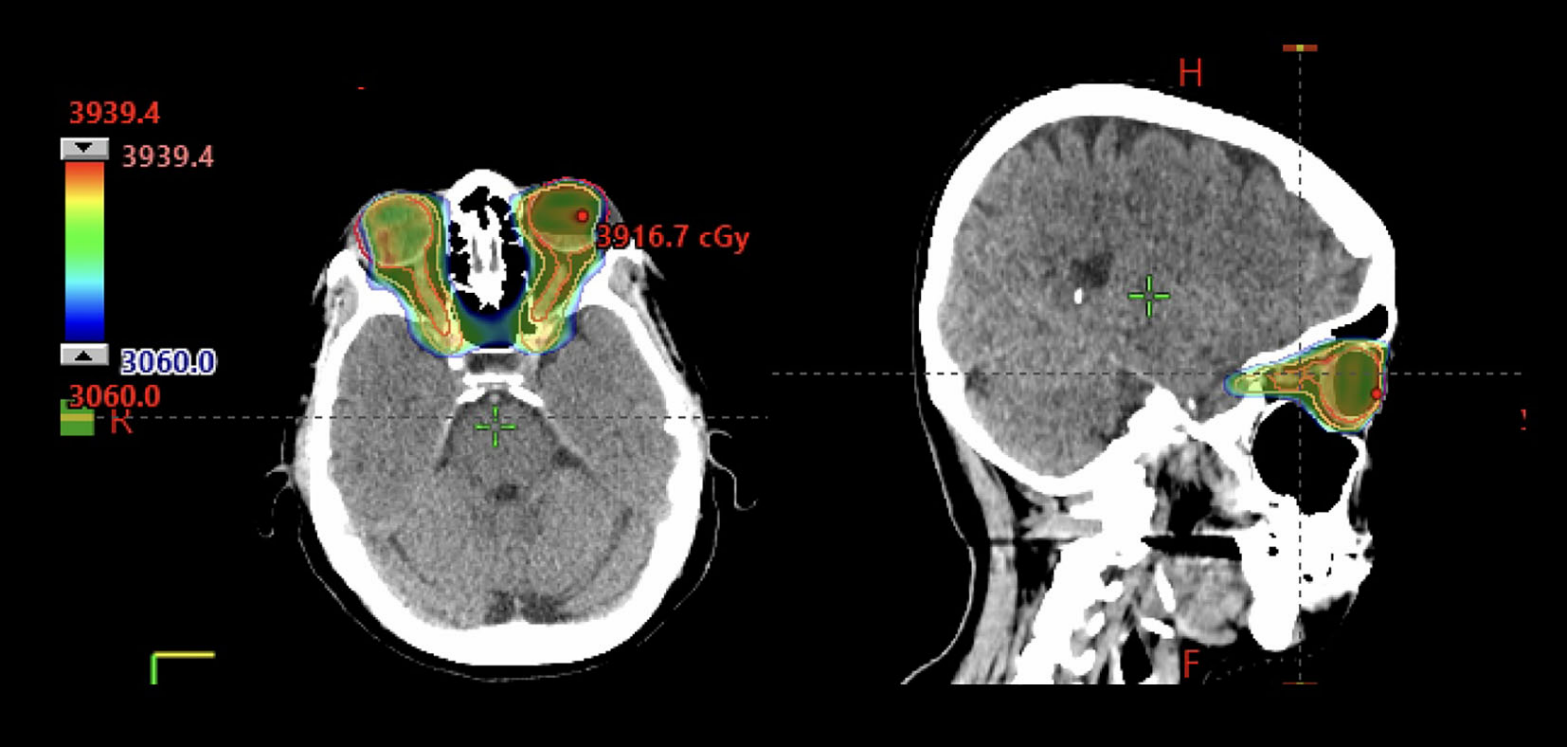
72-year-old immunocompetent woman with bilateral primary vitreo-retinal lymphoma who received involved site radiotherapy to the bilateral orbits and optic nerves to 36 Gy in 20 fractions using VMAT.

In general, RT target volumes for PCNSL should be comprehensive and include the whole brain even for focal parenchymal disease given the high risk of dissemination. Patients with PCNSL also have a high risk of vitreoretinal involvement, thus there is a low threshold for including the posterior chamber in target volumes. For relapsed/refractory disease, RT can be incorporated in the palliative or bridging setting, specifically with the goal of either cytoreduction or temporal bridging to treatments such as to CAR T-cell therapy. RT has been shown to have promising cytoreductive capability in this context, and one can consider focal RT with a hypofractionated approach (i.e., 30 Gy in 10 fractions) given refractory disease more often involves the primary site of disease (23) and there is typically a time restriction for treatment delivery (4). This is a relatively new area of investigation and further study in this area is ongoing. In the salvage setting without plans for definitive systemic therapies (i.e., AHCT or CAR T-cell therapy), 23.4 Gy-36 Gy WBRT plus boost to gross disease of 36-45 Gy can be considered (**Figure 1**). Single institutional experience reports safety of reduced-dose WBRT followed by boost delivered as single-fraction stereotactic radiosurgery to a median of 12 Gy (range, 12-15Gy) (24). For patients presenting with primary intraocular lymphoma, RT to 30.6-36 Gy in 17-20 fractions to bilateral globes and optic nerves to the level of the chiasm should be delivered without whole brain coverage (**Figure 2**); however, 60-90% of patients experience relapse in the brain parenchyma, so untreated brain tissue should be monitored closely (25).

### Secondary CNS lymphoma

For secondary CNS lymphoma, management is similar in many regards to PCNSL; namely, definitive treatment generally requires chemotherapy, AHCT, and/or CAR T-cell therapy (26, 27). The Bruton’s tyrosine kinase inhibitor (BTKi) ibrutinib may be considered as a single-agent therapy (28). The largest prospective study for SCNSL was the phase II MARIETTA trial, which demonstrated a 75% overall response rate after immunochemotherapy (sequential combination of rituximab, methotrexate, cytarabine, thiotepa [MATRix] and rituximab, ifosfamide, carboplatin, and etoposide [RICE] followed by AHCT) and a 2-year progression-free survival of 71% in patients with *de novo* SCNSL. RT can be considered as bridging therapy prior to AHCT or CAR T-cell therapy, or as a component of salvage therapy. Understanding systemic disease status at the time of addressing CNS involvement can help guide CNS-directed RT volumes and dosing. Despite most patients having been heavily pre-treated with poor prior CNS disease response to chemotherapy, multiple series have shown promising cytoreduction and palliation with RT. In one series of 44 patients with SCNSL, RT achieved an objective response rate of 88% and clinical improvement rate of 76%; 31% of patients were still alive at 8 years post-RT (29). One series showed no significant neurologic toxicity with a mean dose of 30 Gy and symptom improvement in 10/10 symptomatic patients. In another study of 58 patients with progressive SCNSL referred for RT, most received WBRT (86%) with a median dose of 30 Gy (IQR 24-30) over 10 fractions, and among patients for whom RT was successfully used to bridge to additional therapy, 29% achieved long-term survival (30).

**Figure 3:**
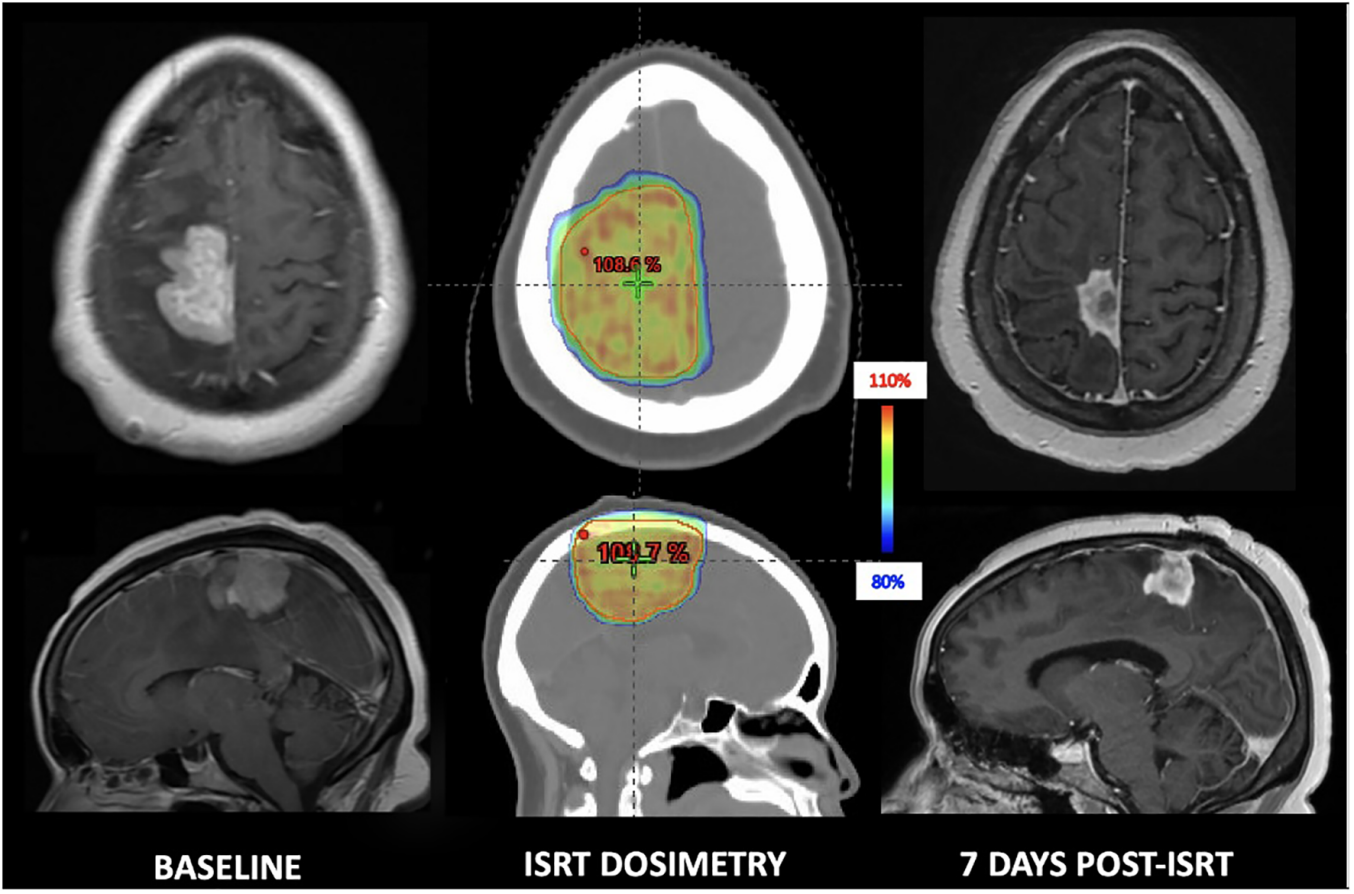
29-year-old immunocompetent woman with secondary CNS lymphoma who underwent focal bridging to 33 Gy in 11 fractions and achieved a swift partial response prior to CAR T-cell therapy.

Therefore, interest is shifting to using RT as a bridge to novel therapeutics, but dosing and target volumes are highly varied. At this time, the only consensus in the use of RT in this setting is that RT should be stopped prior to CAR T-cell therapy and that comprehensively treating gross disease when possible is associated with improved outcomes (31). In a series with 12 patients with CNSL receiving RT as bridge to CAR T-cell therapy, 8 achieved complete response (CR), 1 partial response (PR), and 1 had progressive disease (PD) (32). Although data are limited, initial studies have not seen associations between RT and elevated risk of ICANS (4, 33, 34). Future study is indicated. We favor a focal RT approach to a dose of 30-33 Gy in 10-11 fractions when bridging (**Figure 3**).

**Figure 4:**
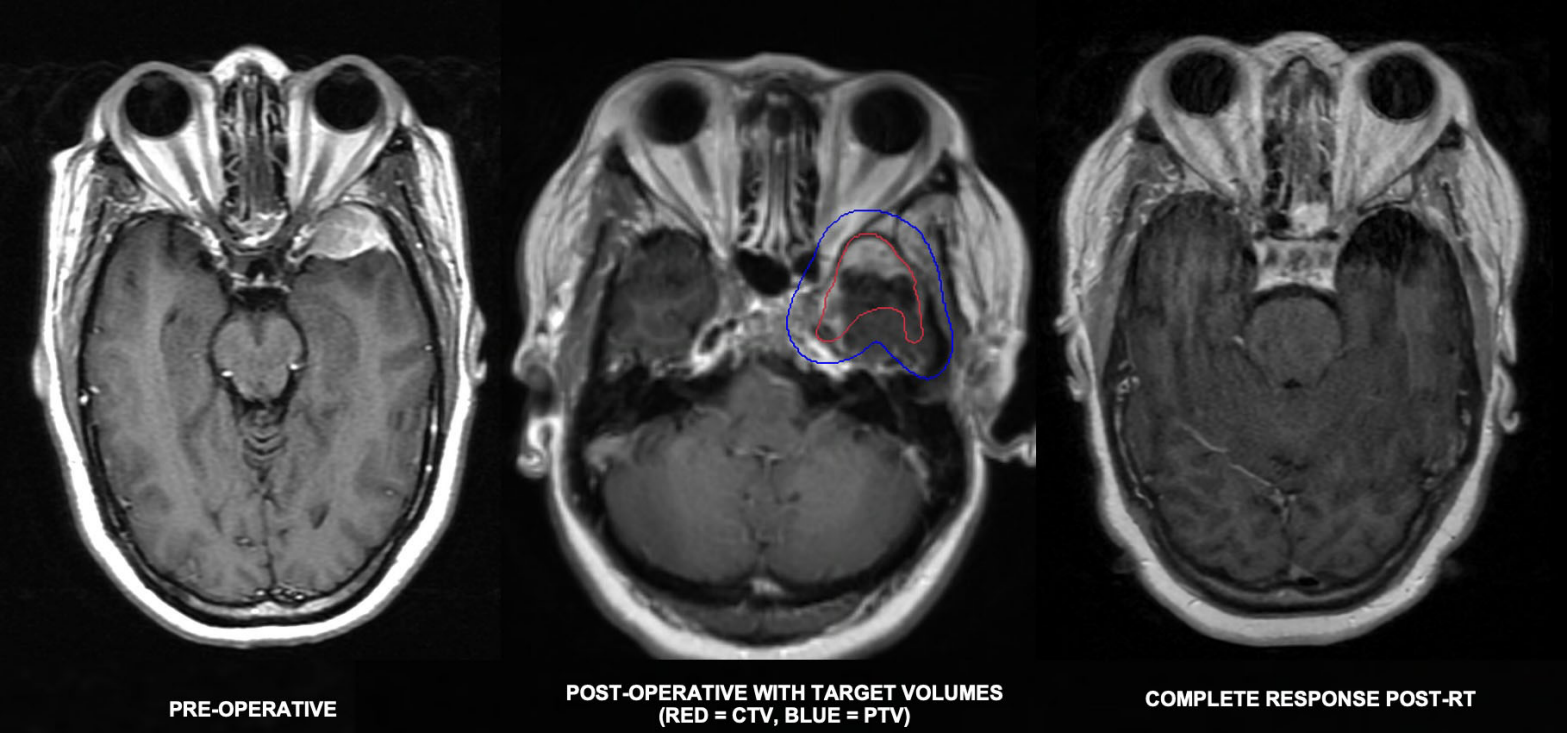
48-year-old woman who presented with headaches found to have a 3.4cm dural-based left sphenoid wing mass initially thought to be meningioma based on imaging appearance. He underwent partial resection with pathology revealing marginal zone lymphoma. Staging PET and blood peripheral flow cytometry were negative for disease involvement. He completed 24 Gy in 12 fractions and achieved a complete response.

Marginal zone lymphoma can involve the dura, which can be treated with systemic therapy alone (e.g., single agent rituximab if CD20+) (35) with consideration of focal RT versus WBRT depending on extent of involvement to a dose of 4-24 Gy in 2-Gy fractions (**Figure 4**) (36, 37). Mantle cell lymphoma can rarely involve the CNS (<5%) but is associated with dismal prognosis. Data suggest that novel therapeutics such as Bruton’s tyrosine kinase inhibitors may be preferred over standard CNS-penetrant systemic therapy in mantle cell lymphoma, demonstrating prolonged overall survival and time to CNS progression with ibrutinib (38). RT dosing is varied, but very low-dose RT (4 Gy in 2 fractions) has been shown to be effective in heavily treated relapsed, refractory systemic disease; (39) therefore, it can be considered in the CNS setting (40).

### Multiple myeloma or plasmacytoma

CNS involvement of multiple myeloma (MM) is seen more often in younger patients (median age 50 years versus typical median age of MM onset 65-70 years) and those with IgA subtypes (27% of patients with CNS involvement versus 1-2% with IgD and biclonal subtypes), deletions of 13q and 17, or elevated lactate dehydrogenase (41). CNS involvement is more often seen in the secondary setting with a short median time to onset of only 2 years from initial diagnosis, and only 22% of cases have CNS involvement at diagnosis. In addition to imaging, CSF cytology with flow cytometry should be performed, especially given that polyclonal plasma cells are not specific to MM and leptomeningeal involvement has been reported in up to 57-59% of CNS myeloma cases (41, 42). Apart from direct CNS involvement, neurologic emergencies from myelomatous disease are often a result of osseous lesions, such as spinal cord compression or cranial nerve deficits from base of skull involvement. Ocular involvement is very rare, but patients with visual symptoms should be evaluated by an ophthalmologist given ocular complications of plasma cell dyscrasias, such as accumulation of M-protein and paraneoplastic syndromes, have been reported (43).

In the emergent setting with neurologic signs or symptoms, early initiation of steroids is critical. There is no standard approach to the treatment of CNS myeloma; however, of the limited published data, a multi-modality approach is favored (41, 44, 45). Systemic therapy is the backbone of treatment, importantly a regimen that can cross the blood brain barrier and have fast onset, as these patients (with or without RT) have been shown to have superior outcomes to those without systemic therapy. IT therapy has not been shown to be effective as a monotherapy. In one study, long-term survivors of CNS myeloma had received combined modality therapy including RT, IT chemotherapy, and immunomodulatory agents (45).

**Figure 5:**
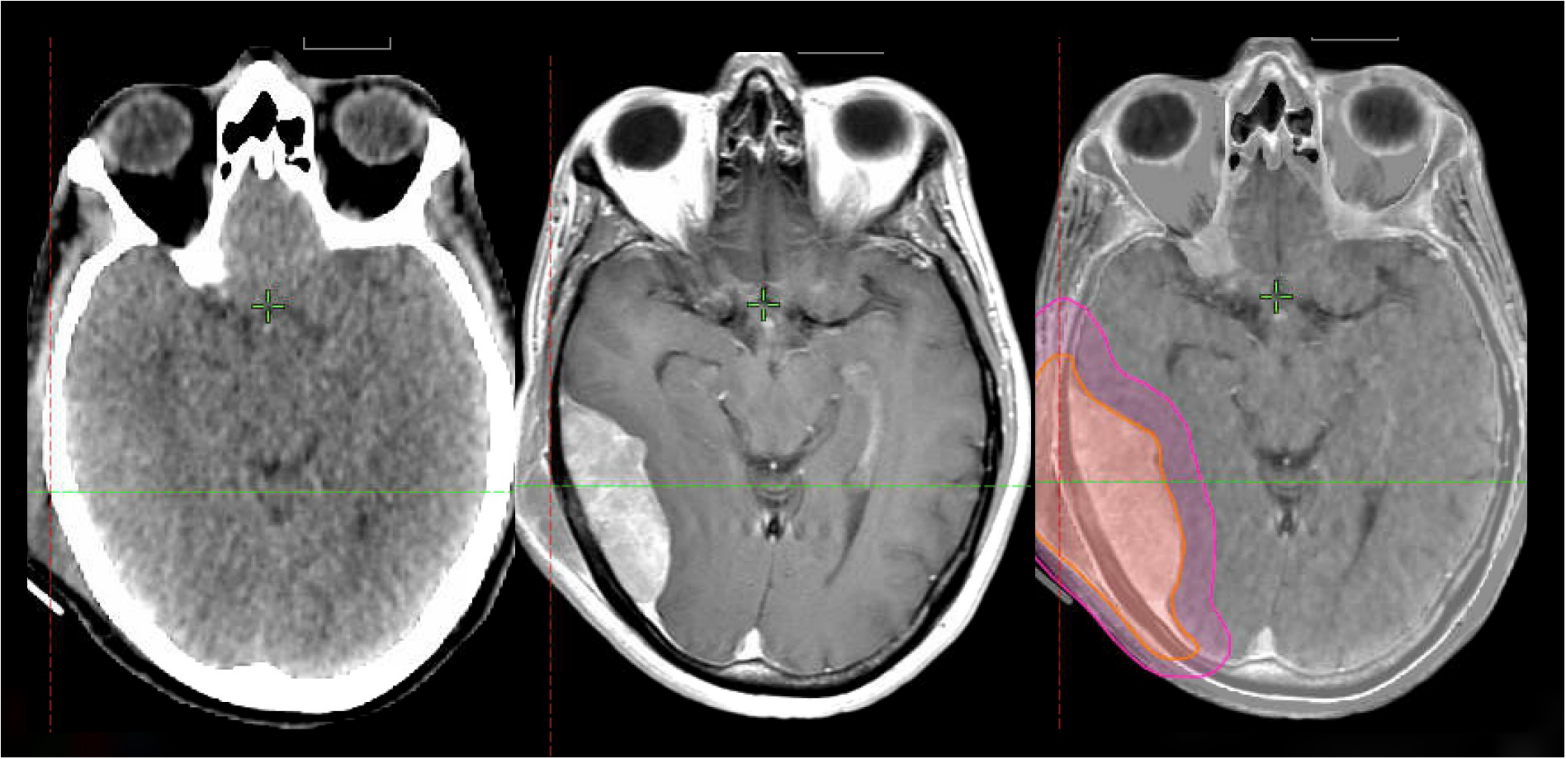
50-year-old man with R-ISS 1 multiple myeloma with a right scalp myelomatous lesion status-post-transplant while on maintenance therapy. MRI showed osseous and dural involvement. He received 24 Gy in 12 fractions with VMAT and achieved a complete response.

For patients with focal deficits and without evidence of leptomeningeal involvement, a focal RT field can be implemented, ideally with IMRT or other conformal techniques to spare normal bone marrow in these patients at particularly high risk of cytopenias (**Figure 5**). While individual- and regimen-dependent, often RT can be given in conjunction with biologic agents used in multiple myeloma (e.g., bortezomib, daratumumab); however, with limited data on bispecific antibodies, care should be given when used with RT concurrently given the theoretical increased risk of immunogenicity (46). For patients with overt spinal cord compression with neurologic deficits, we prefer long (i.e., 24-30 Gy in 2-3-Gy fractions) as opposed to short course (i.e., 20 Gy in 5 fractions) given improved motor function outcomes (47). If the patient has very poor performance status or if treatment needs to be completed in a short time span, one can consider 15-20 Gy in 5 fractions. If a single fraction needs to be given, one can consider 8 Gy in 1 fraction; however, a fractionated approach is preferred in these patients when possible. Heavily pretreated patients tend to have more refractory disease; thus, they may require higher doses or more prolonged courses to achieve durable disease control. Similarly, patients with solitary plasmacytoma may require higher cumulative doses for durable response, for instance 36-50 Gy in 2-Gy fractions (48). In the emergent setting, one can consider starting with a hypofractionated approach (3-4 Gy per fraction) then transition to more protracted, definitive dosing after initial response. For patients with leptomeningeal disease involvement on imaging or CSF, CSI should be considered with doses ranging from 20-30 Gy in 2-Gy fractions. In one study, 4/9 patients who achieved long-term survival received CSI of 20-30 Gy within the context of multi-modality therapy (49).

### Leukemia

For patients with symptomatic CNS leukemia, RT should be considered especially when other CNS-directed therapy has failed. (16) For patients undergoing potentially curative allogeneic transplant, additional RT to the CNS can be considered for patients with acute lymphoblastic leukemia (ALL) with history of CNS involvement or for patients with particularly high-risk disease (e.g., T-cell), and for acute myeloid leukemia (AML) with CNS involvement. Pre-transplant conditioning regimens including CNS-directed RT improved 5-year recurrence-free survival for CNS-involved AML comparable to those without CNS involvement (32 vs 35%, respectively), both notably higher than the 6% 5-year recurrence-free survival seen after IT chemotherapy (50). Choice of comprehensive (i.e., CSI) versus focal RT to the CNS depends on the patient’s clinical status and expected long-term outcomes, but comprehensive CSI is favored by many in the curative setting, though some prefer whole brain boost, and prospective data comparing these strategies do not exist (51).

**Figure 6:**
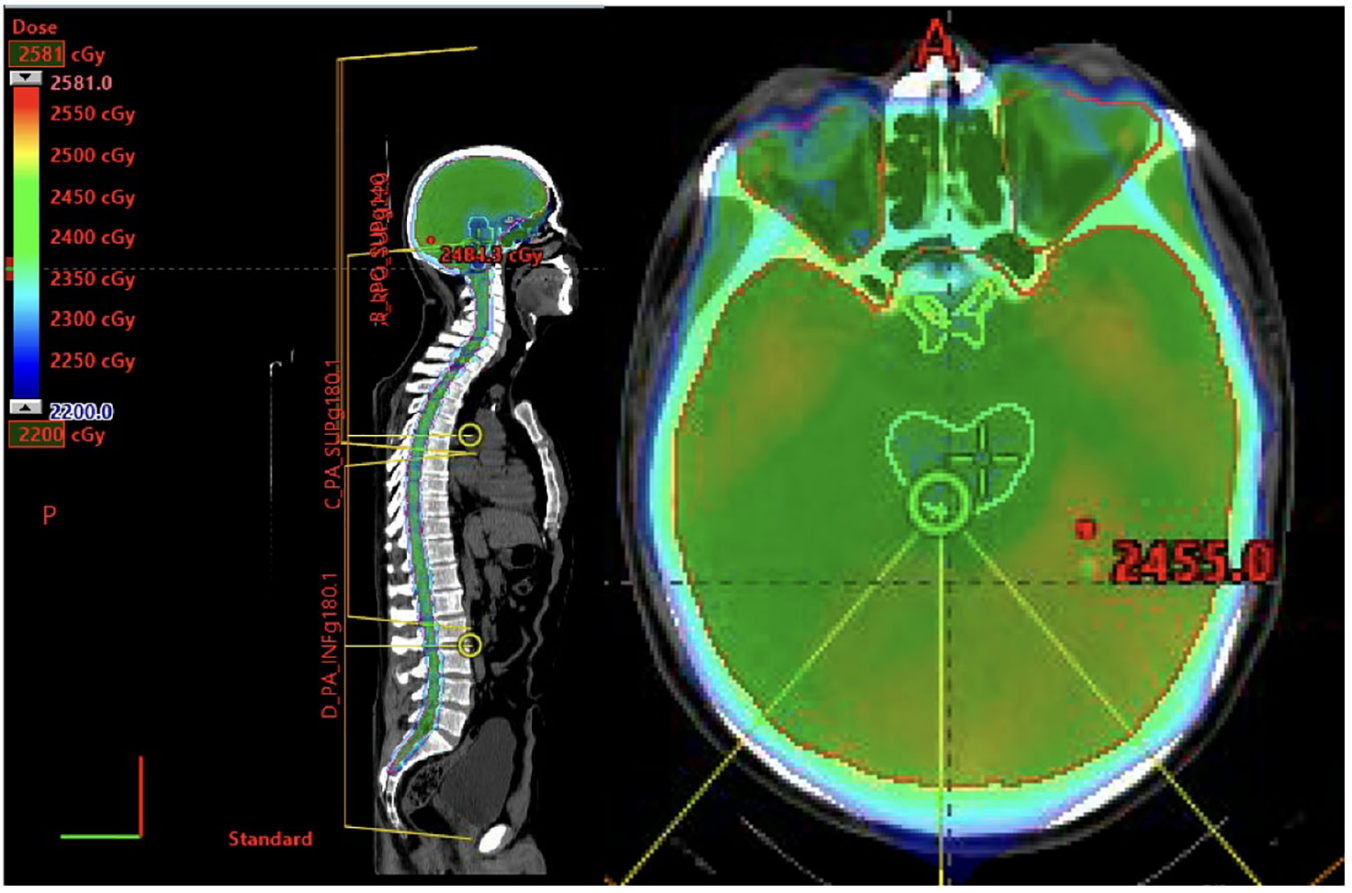
42-year-old Male with Philadelphia chromosome positive B-cell ALL s/p hyper CVAD, TKI, and full-dose TBI followed by stem cell transplant. One year later he experienced an isolated CNS relapsed diagnosed on lumbar puncture and MRI showing radiographic optic nerve involvement. He cleared his CSF s/p hyper-CVAD and IT cytarabine. He received consolidative RT with proton craniospinal irradiation to 23.4 Gy in 13 fractions.

These patients are at high risk for vitreoretinal involvement; therefore, the posterior chamber and optic nerve should be covered for all cases (**Figure 6**). Target volumes should be more comprehensive (i.e., CSI) as the limited data have all shown improved outcomes with larger fields. In a pediatric cohort (among whom 40 of 41 were in complete remission at time of RT), there was a trend toward improved disease-free survival with CSI versus WBRT (52). In a cohort of 163 patients, those treated with WBRT or CSI had better 12-month CNS-progression-free survival compared to those treated with focal RT (77% vs 51%, respectively) (53).

**Figure 7:**
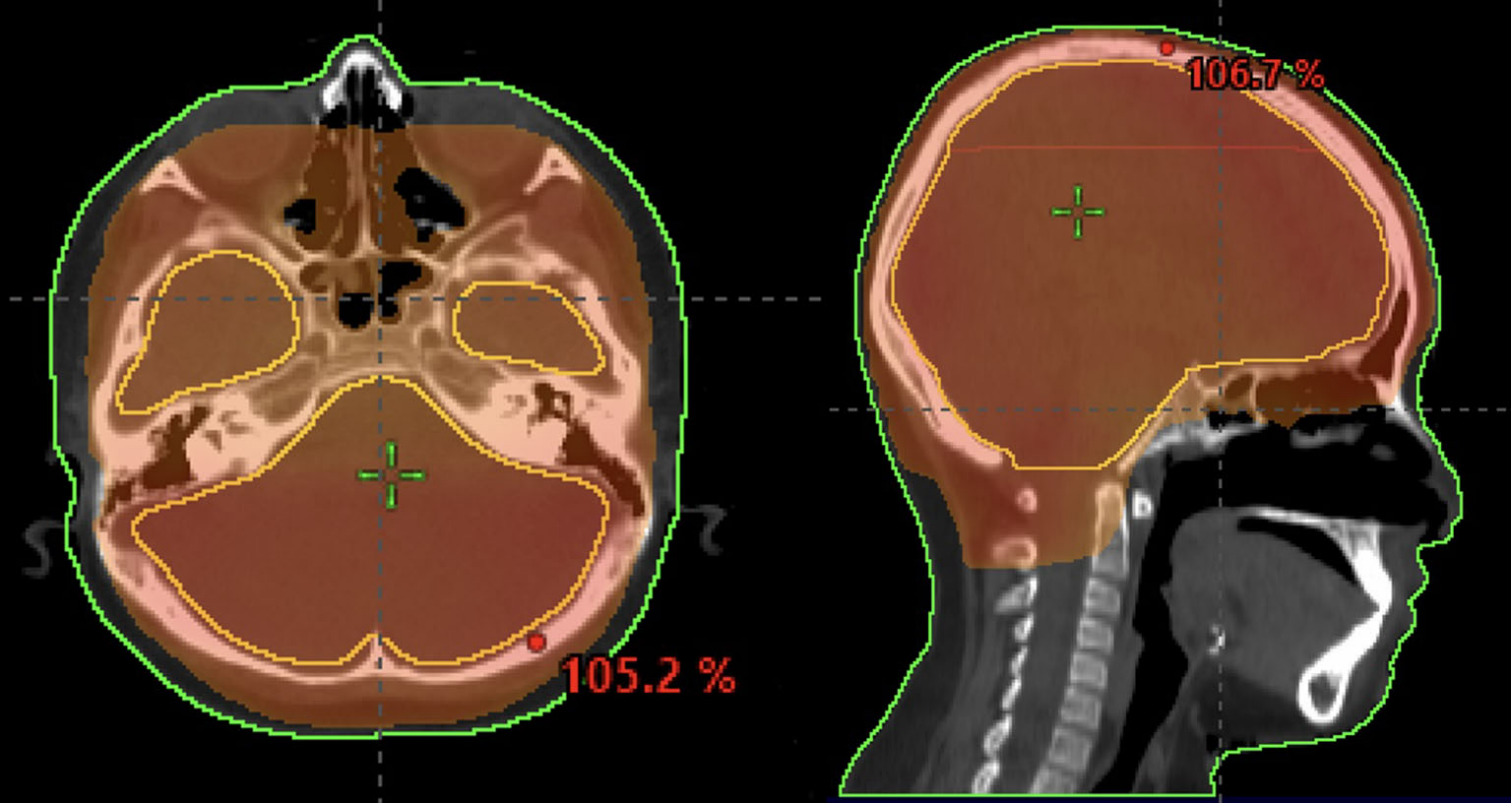
8-year-old patient with leukemia who received a whole-brain radiotherapy boost to a dose of 18 Gy. The 95% isodose line is shown.

Per the International Lymphoma Radiation Oncology Group (ILROG) guidelines, a dose of 18-24 Gy delivered comprehensively (i.e., CSI) should be considered in patients with CNS relapse or prior to allogeneic transplant. When CSI is planned for patients who are to receive a myeloablative conditioning regimen involving total body irradiation (TBI), the CSI dose should be factored into the TBI dose and the total cumulative CSI dose should not exceed 24 Gy. At University of California San Diego, CNS-directed RT is recommended for CNS 2-3 B-cell ALL (i.e., white blood cell [WBC] count <5/mL with blasts in CSF or WBC is ≥5/mL with blasts in CSF or clinical signs of leukemia in the CNS) and in all patients with T-cell ALL who have CNS disease. In general, CSI is preferred to WBRT unless the patient has CNS 1 T-cell ALL (i.e., no blasts in the CSF) or if the CSF has cleared in response to chemotherapy (**Figure 7**).

We recommend CSF sampling prior to RT for all patients to assess response to systemic and IT therapies. MRI evaluation prior to RT is recommended in patients with prior MRI abnormalities or no previous MRI. For CSF-only involvement in patients with complete response to chemotherapy, we generally deliver a cumulative dose of 18 Gy WBRT (i.e., 6 Gy WBRT boost accounting for TBI dose typically of 12 Gy). For patients with radiographic leptomeningeal disease or cranial neuropathies at diagnosis or relapse, our general practice is to add 6-12 Gy CSI in addition to TBI (for a cumulative of 18-24Gy to the craniospinal axis).

When planning CSI, bone-marrow and abdominal compartment-sparing techniques should be explored, namely proton therapy or volumetric modulated arc therapy (VMAT), since these patients often have poor marrow reserve or are planned for radiation dose exposure to their gastrointestinal tract (i.e., TBI). Cumulative lung dose should also be carefully evaluated in patients preparing to undergo transplant as mean lung dose is correlated with peri-transplant mortality (54). As noted above, the vertebral bodies should be considered an OAR for children, and particular attention should be paid to vertebral body dose distribution given risk of scoliosis and impaired growth that can be seen at doses of even 10-20 Gy (55). At Stanford University, for skeletally immature patients, the brain is typically boosted to an additional 12 Gy (24 Gy total), while the spine receives a 6 Gy boost (18 Gy total); skeletally mature patients receive a 12 Gy CSI boost.

Chloroma can be treated to 24 Gy in 12 fractions with a gross tumor volume (GTV) plus a 0.5-1 cm margin for clinical target volume (CTV). In male patients with CNS leukemia, providers should ensure that a testicular ultrasound was done as testicle-directed RT should be delivered in those with involvement. In the absence of a testicular ultrasound examination in patients with T-cell ALL, consolidation with testicular RT should be considered. Some institutions routinely utilize prophylactic testicular boost to 4 Gy in all male leukemia patients or all male patients with ALL, while others reserve this for patients with history of testicular leukemia, and this can be considered on a patient-by-patient basis (56).

## Discussion

Treatment of hematologic malignancies requires a multidisciplinary approach. CNS involvement adds another layer of complexity given the challenge of ensuring CNS penetrance with systemic therapies, aggressive or resistant genetics, need for rapidity of response in the setting of neurologic symptoms, and concern for toxicity. While historically RT played a central, if not exclusive, role in treating CNS hematologic malignancies, in the modern era, concerns for neurotoxicity from high-dose comprehensive RT treatments amidst the promise of novel, targeted agents have led to a narrower role for RT. In the setting of urgent neurologic compromise from hematologic malignancies, the role of RT is of paramount importance as it can be safely and promptly delivered in coordination with a multimodality approach. Radiation oncologists can play a critically important role if we are involved early and coordinate with our colleagues for safe and effective RT delivery.

Treatment-associated neurotoxicity is a concern for any CNS-directed therapy. These patients often have multifactorial neurologic or cognitive compromise from disease burden and prior CNS-penetrant systemic therapies. For instance, high-dose IV methotrexate can have a variety of complications such encephalopathy, delayed multifocal leukoencephalopathy, or even acute transverse myelitis (57). With regards to RT, neurotoxicity has been shown to be associated with dose and treatment volume. When combined with CNS-penetrant systemic agents, significant neurotoxicity has been observed when used both concurrently and prior to cytotoxic therapy, partly due to enhanced permeability of the blood brain barrier likely leading to increased concentration of systemic agents in CSF (58). Historically, standard doses for WBRT (often 40 Gy or higher) were associated with significant neurotoxicity; however, a reduced-dose WBRT approach (23.4 Gy in 13 fractions) has been prospectively shown as part of combination therapy to have excellent disease control without significant neurocognitive decline (22, 59, 60). Final analyses of neuropsychological testing from the maturing RTOG 1114 trial data are eagerly anticipated. Beyond RT dose, treatment volume incorporating critical structures (i.e., hippocampus) can also impact neurocognition (61–63). Therefore, establishing a better understanding of appropriate clinical scenarios where focal RT with smaller field sizes can be effectively implemented is warranted. The applicability of hippocampal-sparing WBRT and memantine for memory preservation in hematologic malignancies is an unanswered question, but this approach has not been implemented by the authors of this review. In pediatric patients, use of memantine is still under active investigation on the Children’s Oncology Group protocol ACNS 2031, albeit for RT in primary CNS tumors. In the era of novel therapies, perhaps this dogma of universally comprehensive RT in CNS hematologic malignancies may not be as applicable, and we can consider building off the principles of involved site radiotherapy (ISRT) now established as standard for extracranial lymphomas (64).

**Figure 8:**
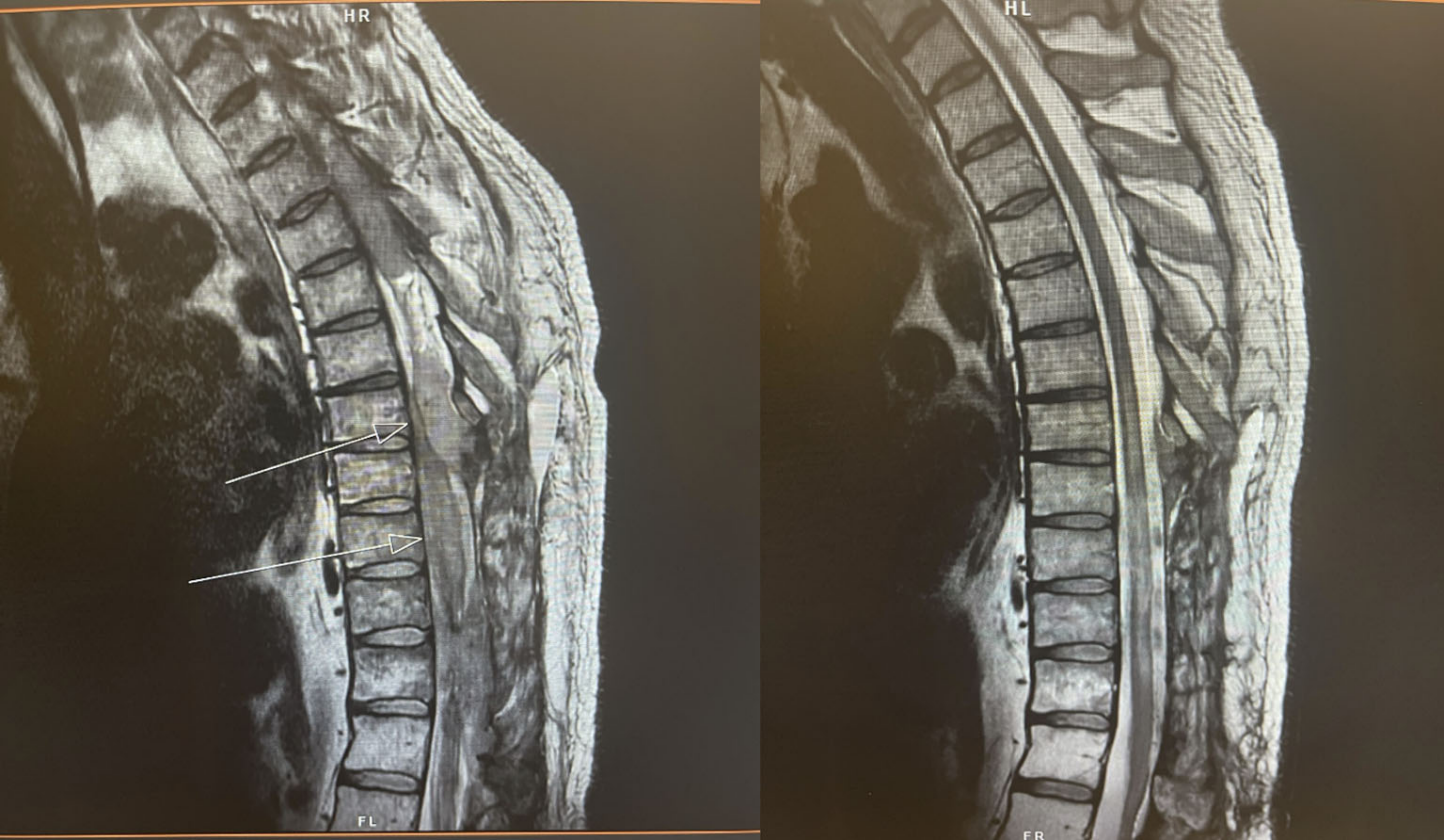
45-year-old man with heavily pretreated refractory DLBCL causing cord compression and paralysis. He was initially recommended for hospice and referred to radiation oncology for consideration of palliative RT. (L) before and (R) 3 days after completion of thoracolumbar RT to 20 Gy in 5 fractions. Following excellent response to RT, patient became a candidate for CAR T-cell therapy.

There is much excitement building around novel therapeutics (e.g., CAR T-cells, bispecific antibodies) and the possibility of a durable response in otherwise challenging, refractory scenarios. Converting patients with progressive or refractory disease via cytoreductive bridging is a promising opportunity for RT in the modern era. Preliminary data limited to small cohorts suggest that the combination of CNS RT with novel targeted agents and cellular therapies may be safe and tolerable (4, 30, 65). In addition, we have experience with multiple patients whose disease was thought to be overwhelmingly refractory to systemic therapy, and the consultation for these patients was for palliative RT prior to hospice. In several of these patients, RT produced such a powerful disease control and symptomatic benefit that the patient was re-evaluated and deemed appropriate to proceed with CAR-T cell therapy (**Figure 8**). These cases serve as an important reminder that unlike many solid tumor histologies or primary CNS tumors, many patients with CNS involvement of hematologic malignancies may have a temporary poor performance status that is reversible, and RT can remain effective even in some of the most chemorefractory patients (66). Therefore, patient performance status in isolation is not a reliable indicator of prognosis in CNS hematologic malignancies. For bridging RT to CAR T-cell therapy, we favor focusing on bulky or symptomatic sites of disease with a focal field (i.e., GTV with a 1cm margin to CTV) to a dose of 30-33 Gy in 10-11 fractions. However, the appropriate dose and target volume are not yet defined. For instance, the role of CSI prior to CAR T-cell therapy is not established, leaving the question of how best to bridge patients with leptomeningeal dissemination open. When considering the extent of the target volume, one must consider risk of neuraxis dissemination and whether the neuraxis can be adequately treated with non-RT approaches such as CNS-penetrant or IT therapies. These unanswered questions warrant thorough histology-specific patterns-of-failure analyses after focal RT, but one can see the potential benefit of a focal, dose-intensified approach for refractory disease as a bridge to comprehensive therapy.

## Conclusions

RT is a powerful tool for achieving quick responses in hematologic malignancies and therefore should be considered early in urgent neurologic settings. Thorough workup and discussions with the multi-disciplinary team are critical to best incorporate RT in the context of other CNS-penetrating therapies. Further work is warranted on defining RT target volumes and doses in the context of novel therapeutics.
